# Artificial intelligence in veterinary education: self-perceived knowledge, use, and attitudes among veterinary students in Spain and Portugal

**DOI:** 10.3389/fvets.2026.1816115

**Published:** 2026-06-17

**Authors:** Ana Lourdes Oropesa, Santiago Mendo-Lázaro, Antonio Gonzalez, Benito León-Del-Barco, José Antonio Tapia

**Affiliations:** 1 Toxicology Area, Department of Animal Health, Faculty of Veterinary Sciences, University of Extremadura, Cáceres, Spain; 2 Department of Psychology and Anthropology, Faculty of Teacher Training, University of Extremadura, Cáceres, Spain; 3 Department of Physiology, Faculty of Veterinary Sciences, University of Extremadura, Cáceres, Spain

**Keywords:** AI literacy, artificial intelligence, curriculum integration, digital engagement, health professions education, veterinary education

## Abstract

Artificial intelligence (AI), especially generative AI and large language models, is increasingly influencing higher education and health professions training. However, there is still limited empirical evidence about what veterinary students know about AI, how they use it, and how they perceive it. The aim of this study was to evaluate self-perceived AI-related knowledge, use, and attitudes among veterinary students in Spain and Portugal, and to analyze the influence of institutional context, prior AI training, and digital engagement. A cross-sectional survey was conducted during the 2023–2024 academic year with 340 undergraduate and postgraduate veterinary students from public and private institutions in Spain and Portugal. The questionnaire included sociodemographic questions and nine Likert-scale items assessing self-perceived AI knowledge, use, and attitudes. Composite scores were calculated and transformed using the Percentage of Maximum Possible (POMP) method (0–100 scale). Internal consistency of the instrument was high (*ω* = 0.842; *α* = 0.835). Data was analyzed using MANOVA, one-way ANOVA, independent samples t-tests, and Pearson correlation analyses. Significant differences were observed between institutions in self-perceived AI knowledge, use, and attitudes. Students who had received prior AI training showed higher self-perceived knowledge and use scores (*p* < 0.001) and more positive attitudes toward AI (*p* < 0.001). Although formal AI training was limited in many institutions, students with any type of prior exposure—self-directed, university-based, or external—reported greater engagement and felt more prepared to use AI tools. Daily social media use showed a small but statistically significant positive correlation with self-perceived AI knowledge and use (r = 0.115, *p* = 0.034) and with positive attitudes (r = 0.157, *p* = 0.004). Categorized analyses showed a gradual increase in AI-related outcomes with higher levels of digital engagement. Veterinary students in the Iberian Peninsula are already using AI, mainly through informal learning pathways, while structured institutional integration remains uneven. Structured exposure to AI is consistently associated with higher self-perceived knowledge, greater use, and more positive attitudes. These results suggest that deliberate curricular integration and guided AI literacy initiatives are important to prepare future veterinarians for responsible and effective use of AI technologies.

## Graphical abstract

**Figure:**
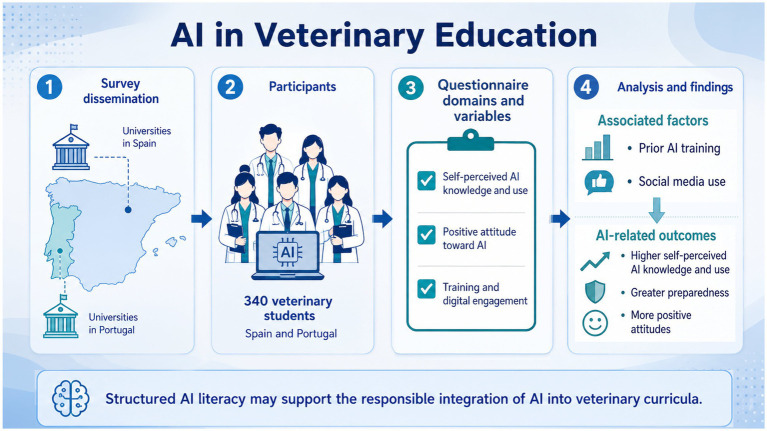
Overview of the cross-sectional study design, questionnaire domains and variables, and main findings regarding self-perceived AI knowledge and use, positive attitudes toward AI, prior AI training, and digital engagement among veterinary students in Spain and Portugal.

## 1 Introduction

In the context of university teaching, artificial intelligence (AI) has moved from a speculative topic to a tangible driver of change in higher education over the past few years. Generative AI, and in particular large language models (LLMs) such as ChatGPT and Gemini, has demonstrated impressive capabilities in text generation, code drafting, and support for complex problem-solving. Universities are increasingly formalizing its use through institutional policies and guidance (1–3).

Generative AI refers to models capable of producing novel content, such as text, images, or other media, by learning patterns from large training datasets (2). LLMs trained on extensive corpora of text and code can generate coherent, context-sensitive responses and adapt to diverse user prompts and tasks (4). Unlike rule-based tutoring systems, these models can flexibly support a wide range of educational activities, from drafting learning materials to answering students’ questions in natural language (5).

Consequently, these AI developments have driven a narrative suggesting that AI could enhance learning efficiency, personalize instruction, and relieve educators of certain routine tasks (6, 7). Despite this optimism, integration across medical disciplines, including veterinary curricula, remains uneven and largely experimental. Most veterinary schools still lack a coherent institutional strategy regarding how AI should be incorporated into teaching, assessment, and competency development. Moreover, empirical research examining what veterinary educators and students know, do, and believe about AI remains limited (8, 9).

In veterinary education, AI should also be considered within the broader ecosystem of anatomical, clinical, simulation-based, and model-assisted teaching resources. Physical and digital anatomical models support the acquisition of spatial understanding, procedural reasoning, and clinical decision-making skills, particularly in anatomy, surgery, diagnostic imaging, and applied clinical training (10). Within this context, AI-based tools may expand the educational value of such models by supporting anatomical interpretation, image-based learning, case generation, adaptive feedback, and interactive 3D or virtual learning environments (11–13). However, these applications also require students to understand both the potential and the limitations of AI systems, including issues related to accuracy, data quality, bias, transparency, privacy, and responsible use (11, 12).

For educators, LLMs offer practical support in lesson planning, case and assessment item generation, and feedback provision, potentially saving time while standardizing certain processes (4, 14). For students, they may function as always-available tutors, providing explanations, reformulations of complex concepts, and simulated clinical scenarios (6, 9). When used rationally and critically, these affordances may provide meaningful educational benefits.

At the same time, the limitations of generative AI are substantial. LLMs are prone to “hallucinations,” responses that are well-phrased but factually incorrect, delivered with unwarranted confidence (4, 15). Concerns also extend beyond accuracy. Scholars and regulators clearly highlight risks related to data protection, algorithmic bias and fairness, and the potential erosion of independent critical thinking if learners over-rely on AI-generated outputs (15, 16).

In this context, a central gap becomes apparent: there is limited empirical evidence describing what veterinary students report knowing about AI, how they are using it in their studies, and which benefits, risks, and practical obstacles they perceive. Although existing work documents both enthusiasm and anxiety surrounding the expansion of AI in veterinary education and practice (5, 6, 16), the perspectives of future veterinarians remain mainly unexplored. The present study addresses this gap by examining veterinary students’ self-perceived knowledge, uses, and perceptions of AI in Spain and Portugal. Specifically, we aim to identify some institutional and individual factors that shape their willingness and capacity to integrate AI responsibly within veterinary curricula.

## 2 Materials and methods

### 2.1 Participants

**Figure 1:**
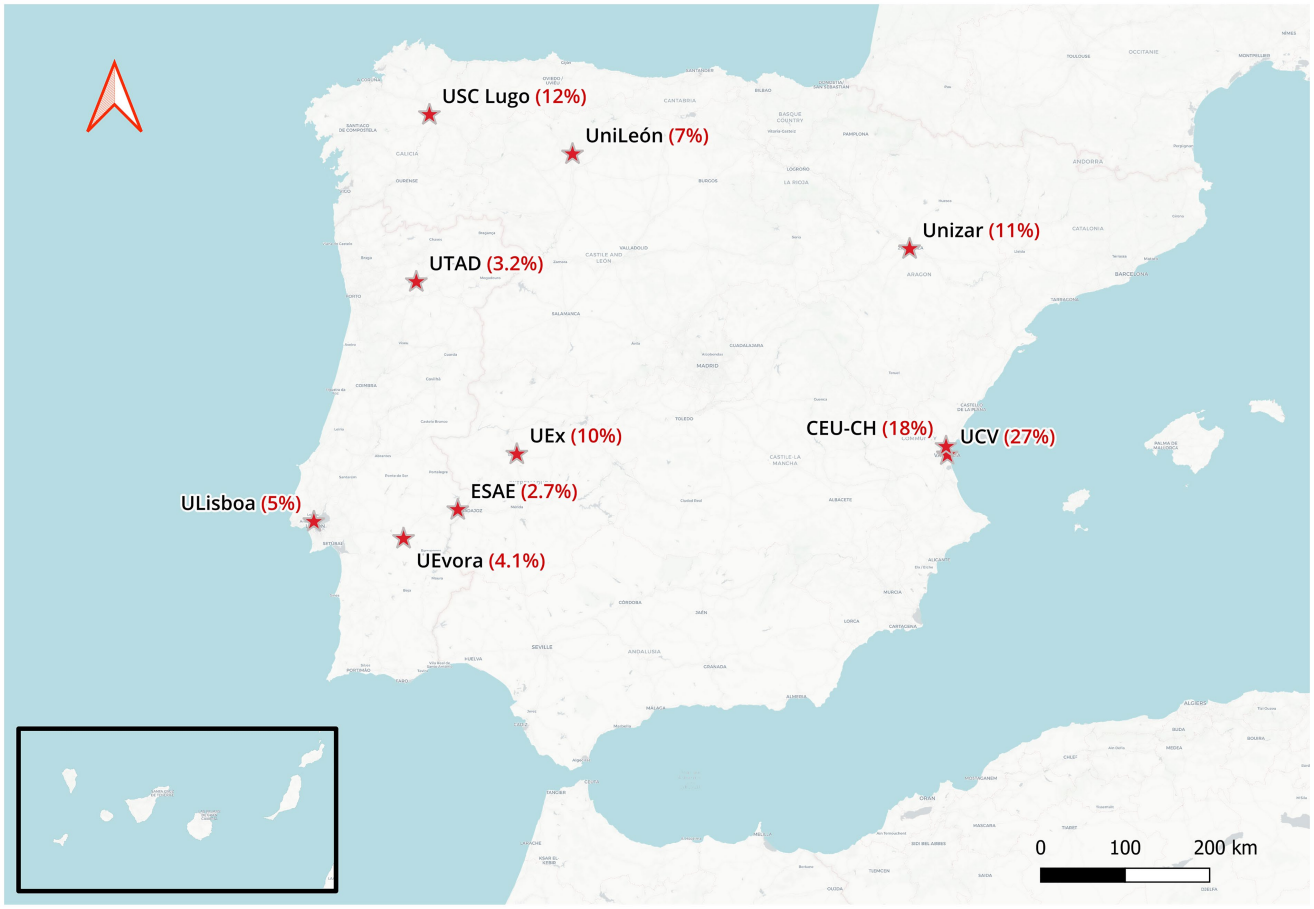
Geographical distribution of participating veterinary teaching institutions in Spain and Portugal. The map shows the institutions from which respondents were recruited and the percentage contribution of each center to the final sample. Percentages indicate the proportion of total respondents from each institution. UCV, Catholic University of Valencia; CEU-CH, CEU Cardenal Herrera University – Valencia; USC Lugo, University of Santiago de Compostela – Lugo; Unizar, University of Zaragoza; UEx, University of Extremadura; UniLeón, University of León; ULisboa, University of Lisbon; UEvora, University of Évora; UTAD, University of Trás-os-Montes e Alto Douro; ESAE, Polytechnic Institute of Portalegre – Escola Superior Agrária de Elvas.

This cross-sectional observational study was conducted during the 2023–2024 academic year among veterinary students enrolled in public and private Faculties/Teaching Centers in Spain and Portugal (Figure 1).

Eligible participants included undergraduate students enrolled in the Veterinary degree program as well as postgraduate (PhD) students holding a Veterinary degree during the study period. Participation was voluntary and based on a non-probabilistic convenience sampling approach. All eligible students who completed the questionnaire during the data collection period were included in the analysis. No prior sample size calculation was performed. Questionnaires with more than 20% missing responses were excluded from the final dataset.

A total of 340 students participated in the study. Of these, 17% identified as male, 82% as female, and 1% as another gender. The predominance of female participants reflects the current demographic composition of veterinary education. Participants’ ages ranged from 19 to 40 years (Median = 23 years).

### 2.2 Questionnaire details

Data were collected using an electronic questionnaire developed in Spanish and English through the Microsoft Forms platform, which allows responses to be exported to Microsoft Excel for further statistical analysis. The survey consisted of 18 items organized into two sections. The first section included nine sociodemographic questions addressing variables such as sex, age, university, country, academic level, previous training in AI, type of AI training received, social media use, and daily hours of social media use. The second section comprised nine items addressing self-perceived knowledge of AI, use of AI tools, and attitudes toward AI. For the statistical analyses, these items were grouped into two composite domains: self-perceived AI knowledge and use (items 1–6) and positive attitude toward AI (items 7–9), as detailed in Table 1.

**Table 1:** Questionnaire items and response options.

Items	Response options
Sociodemographic questions	
Age	Birth date
Gender	Male/female/non-binary
At which University are you studying Veterinary Medicine?	Escola Superior Agrária de Elvas (Instituto Politécnico de Portalegre)/Instituto de Ciências Biomédicas Abel Salazar-Universidade do Porto/Universidad Alfonso X El Sabio – Madrid/Universidad Autónoma de Barcelona/Universidad Católica de Valencia/Universidad CEU Cardenal Herrera – Valencia/Universidad Complutense de Madrid/Universidad de Córdoba/Universidad de Extremadura/Universidad de las Palmas de Gran Canaria/Universidad de León/Universidad de Lleida/Universidad de Murcia/Universidad de Santiago de Compostela – Lugo/Universidad de Zaragoza/Universidade de Évora/Universidade de Lisboa/Universidade Tras-Os-Montes e Alto Douro
Indicate the most advanced course in which you are enrolled in the Veterinary Medicine degree or if you are studying a postgraduate degree related to Veterinary Medicine	First/Second/Third/Fourth/Fifth/Sixth/Postgraduate
In what type of subjects of the Veterinary Medicine degree or postgraduate degree related to Veterinary Medicine do you think it would be most useful to integrate Artificial Intelligence (AI) tools?	Basic subjects (Chemistry, Mathematics, Physiology)/Clinical subjects (Medical Pathology, Surgery, Parasitology)/Subjects related to Food Technology and Hygiene (Food Safety)/Subjects related to Animal Production (Business Management, Ethnology)/Practical subjects (external internships, stays)/Final Project
How many hours a day do you use social media?	I do not use social media/1/2/3/4/5/6 or more
Of the following social media, which one do you use the most?	Facebook/X (formerly Twitter)/Instagram/YouTube/Snapchat/TikTok/other (free text field)
Have you received any type of training (courses, seminars, talks) or do you have information about AI?	yes/no
If you answered ‘Yes’ in the previous question, please specify what type of information or training you have received	Regulated training within the university/regulated training outside the university context/self-taught training
AI-related questions grouped by analytical domain: self-perceived AI knowledge and use (items 1–6) and positive attitude toward AI (items 7–9)	
1. Do you think AI can improve the quality of Veterinary education?	Nothing/something/quite/completely
2. Have you ever used AI-based applications or services in any subject of your Veterinary Medicine degree or postgraduate degree?	Nothing/something/quite/completely
3. Do you think that AI tools would help you to obtain better academic results than you could obtain without them?	Nothing/something/quite/completely
4. Would you feel comfortable using AI systems to improve your educational experience?	Nothing/something/quite/completely
5. Do you think AI will have an impact on the Veterinary profession?	Nothing/something/quite/completely
6. Do you feel yourself prepared to work with AI tools in your future professional activity?	Nothing/something/quite/completely
7. Do you think it is possible to use AI in an ethical and responsible way to complete the tasks and assignments required by your degree?	Nothing/something/quite/completely
8. Would you agree that the use of AI tools to complete school tasks involves cheating or plagiarism?	Nothing/something/quite/completely
9. Would you agree that AI-based tools should be prohibited for completing school tasks in your degree?	Nothing/something/quite/completely

Sociodemographic variables included age, sex, institution, academic level, prior artificial intelligence (AI) training, type of AI training received, and social media use. AI-related questions covered self-perceived AI knowledge, use of AI tools, and attitudes toward AI. For the statistical analyses, these items were grouped into two composite domains: self-perceived AI knowledge and use (items 1–6; raw score range 0–18) and positive attitude toward AI (items 7–9; raw score range 0–9).

Responses in the second section were recorded using a four-point adapted Likert scale (nothing, something, quite, completely), which was recoded from 0 to 3 for composite-score calculation. A four-point Likert-type scale was selected to encourage directional responses and reduce central tendency bias associated with neutral midpoint options. One item was reverse-coded during data processing. Conditional branching logic was applied so that follow-up questions (e.g., type of AI training) were displayed only when relevant.

Composite scores were calculated for the two AI-related analytical domains by summing the corresponding Likert-scale items within each domain and computing the mean composite score for analytical purposes. Specifically, self-perceived AI knowledge and use items yielded a composite score ranging from 0 to 18, and positive attitude items yielded a composite score ranging from 0 to 9. These raw composite scores were used for descriptive analyses and graphical representation where indicated.

To facilitate interpretation and comparability across dimensions with different score ranges, composite scores were additionally transformed using the Percentage of Maximum Possible (POMP) method. The POMP transformation rescales raw scores to a standardized 0–100 metric using the equation:


$$\text{POMP}=\frac{\left(\text{Observed score}-\text{Minimum possible score}\right)}{\left(\text{Maximum possible score}-\text{Minimum possible score}\right)}\times 100$$

This transformation allows effect sizes and group differences to be interpreted on a common percentage scale across figures and analyses.

The questionnaire was developed by faculty members in veterinary science and sociology. Internal consistency of the scale was evaluated using Cronbach’s alpha and McDonald’s omega coefficients. The instrument demonstrated high internal reliability (*ω* = 0.842; *α* = 0.835), based on 336 valid observations after listwise exclusion of three incomplete cases.

The survey was distributed through official institutional channels after contacting the Deans or degree/postgraduate coordinators of all Faculties/Teaching Centers offering veterinary education in Spain and Portugal. Dissemination was carried out through mass, concurrent distribution to all enrolled students at participating institutions. A reminder message was sent several weeks after the initial invitation. The questionnaire remained open from February to May 2024.

### 2.3 Ethical considerations

The first page of the questionnaire provided detailed information about the objectives of the study, procedures, data handling, and participants’ rights. Students were required to provide informed consent by selecting an “I agree” option before accessing the survey. If consent was not provided, the questionnaire could not be completed. Participation was anonymous and voluntary, and respondents could withdraw at any time without penalty. A contact email address was provided for any queries.

Data were processed in accordance with Spanish Data Protection Law 3/2018 and the European Union General Data Protection Regulation (EU Regulation 2016/679). The study protocol was approved by the Bioethics and Biosafety Committee of the University of Extremadura (Spain).

### 2.4 Data analysis and processing

Statistical analyses were performed using IBM SPSS Statistics (version 29) and JASP (version 0.19.3) (17). Likert-scale responses were numerically coded as described above prior to analysis (18).

For graphical representations of categorical distributions (e.g., prior AI training and type of training), proportions were converted into standardized scores ranging from 0 to 100 to represent percentage distributions within each institution. Error bars in figures represent the standard error of the mean (SEM), calculated based on institutional or grouped means as appropriate.

Normality and homogeneity of variance were assessed using the Kolmogorov–Smirnov and Levene’s tests, respectively. Internal reliability of the questionnaire was evaluated using Cronbach’s alpha and McDonald’s omega coefficients. To examine the association between AI-related analytical domains (self-perceived AI knowledge and use, and positive attitude toward AI) and independent variables—including university, country, sex, academic level, previous AI training (and type of training), social media use, and daily hours of social media use—multivariate analysis of variance (MANOVA) was conducted. When appropriate, independent-samples Student’s t-tests were applied for comparisons between two independent groups. An asymptotic two-sided *p*-value < 0.05 was considered statistically significant.

## 3 Results

### 3.1 Inter-institutional differences in self-perceived AI knowledge, use, and attitudes

**Figure 2:**
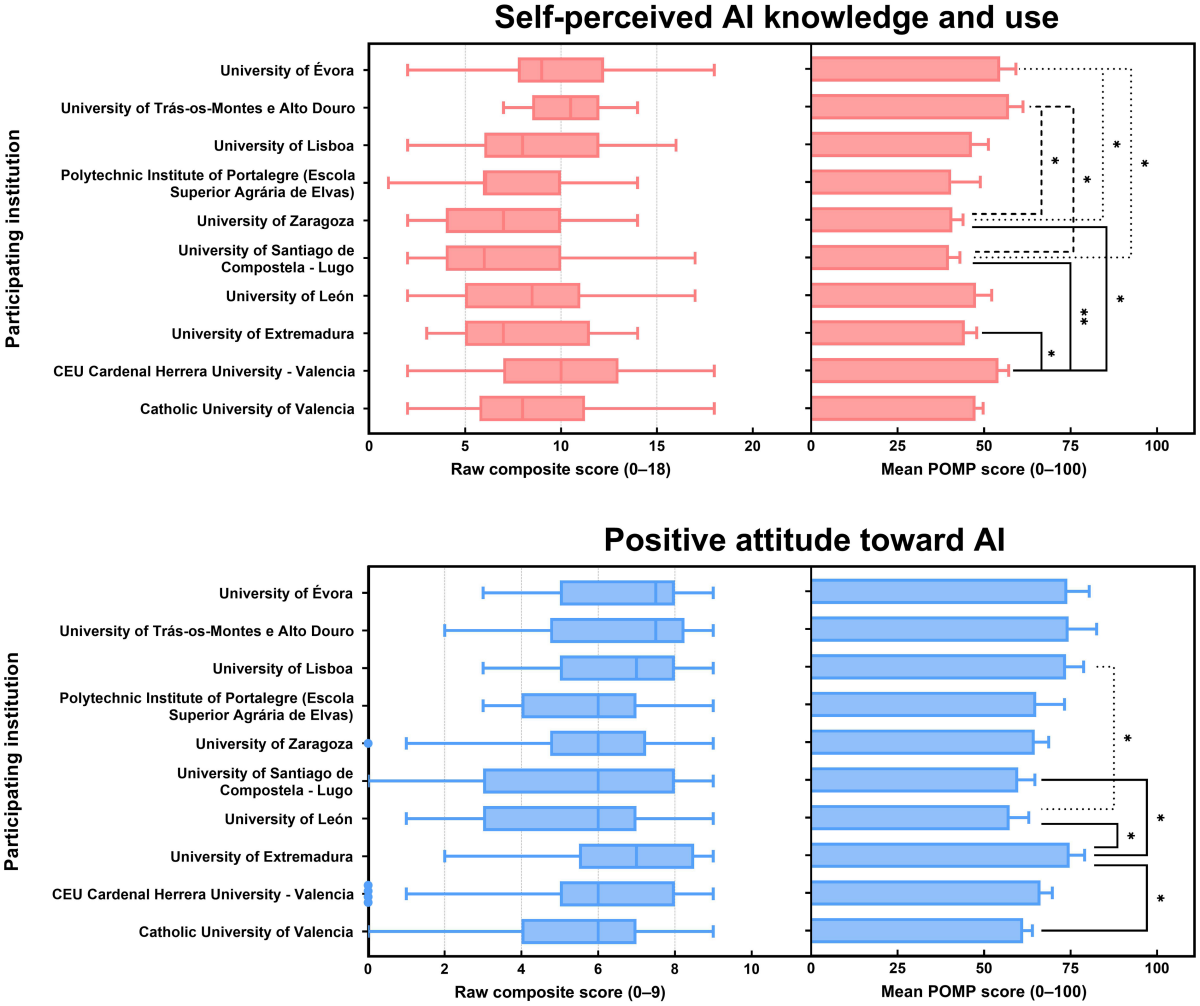
Self-perceived artificial intelligence (AI) knowledge and use (upper panels) and positive attitude toward AI (lower panels) across participating institutions. Left panels display composite mean scores with boxplots (self-perceived AI knowledge and use: 0–18 scale; AI attitude: 0–9 scale). Boxes represent the interquartile range (IQR), the central line indicates the median, and whiskers represent the minimum and maximum values. Right panels show POMP-transformed means (percentage of maximum possible; 0–100 scale). Differences between institutions were analyzed using multivariate analysis of variance (MANOVA), followed by *post hoc* pairwise comparisons. Statistically significant differences are indicated by connecting lines and asterisks (*p < 0.05; **p < 0.01).

The first comparison aimed to determine whether differences existed between students from different institutions regarding self-perceived AI knowledge, use, and attitudes toward AI. Figure 2 illustrates the distribution of composite scores for self-perceived AI knowledge and use, as well as positive attitudes toward AI, across participating universities. Both raw composite scores and Percentage of Maximum Possible (POMP) values are presented.

With respect to self-perceived AI knowledge and use (Figure 2, top, pink bars), variability between institutions was observed. Median composite scores differed across universities, with some centers displaying higher central tendency values and narrower interquartile ranges, while others showed greater dispersion. The POMP-transformed means reflected a similar pattern. *Post hoc* comparisons revealed statistically significant differences between specific institutions. In particular, the highest levels of self-perceived AI knowledge and reported use were observed among students from the University of Évora and CEU Cardenal Herrera University (Valencia), whereas comparatively lower scores were found in students from the Polytechnic Institute of Portalegre (Escola Superior Agrária de Elvas), the University of Zaragoza, and the University of Santiago de Compostela (Lugo). These findings indicate that students’ levels of self-perceived AI knowledge and use were not homogeneous across centers and countries.

Regarding positive attitudes toward AI (Figure 2, bottom, blue bars), composite scores also varied between universities. Although median values generally clustered within a moderate-to-high range, clear inter-institutional differences were evident. Again, the POMP scores confirmed this variability, and several statistically significant pairwise differences were identified between universities. Specifically, the most positive attitudes toward AI were observed among students from the University of Évora and CEU Cardenal Herrera University (Valencia), whereas comparatively lower attitude scores were found in students from the Polytechnic Institute of Portalegre (Escola Superior Agrária de Elvas), the University of Zaragoza, and the University of Santiago de Compostela (Lugo). These differences indicate that students’ perceptions of AI are not uniform across institutions.

Overall, the results indicate that both self-perceived AI-related knowledge/use and attitudes toward AI differ across participating institutions, with statistically significant contrasts observed in multiple inter-university comparisons.

### 3.2 Differences in self-perceived AI knowledge, use, and attitudes according to sex, country, and academic level

**Figure 3:**
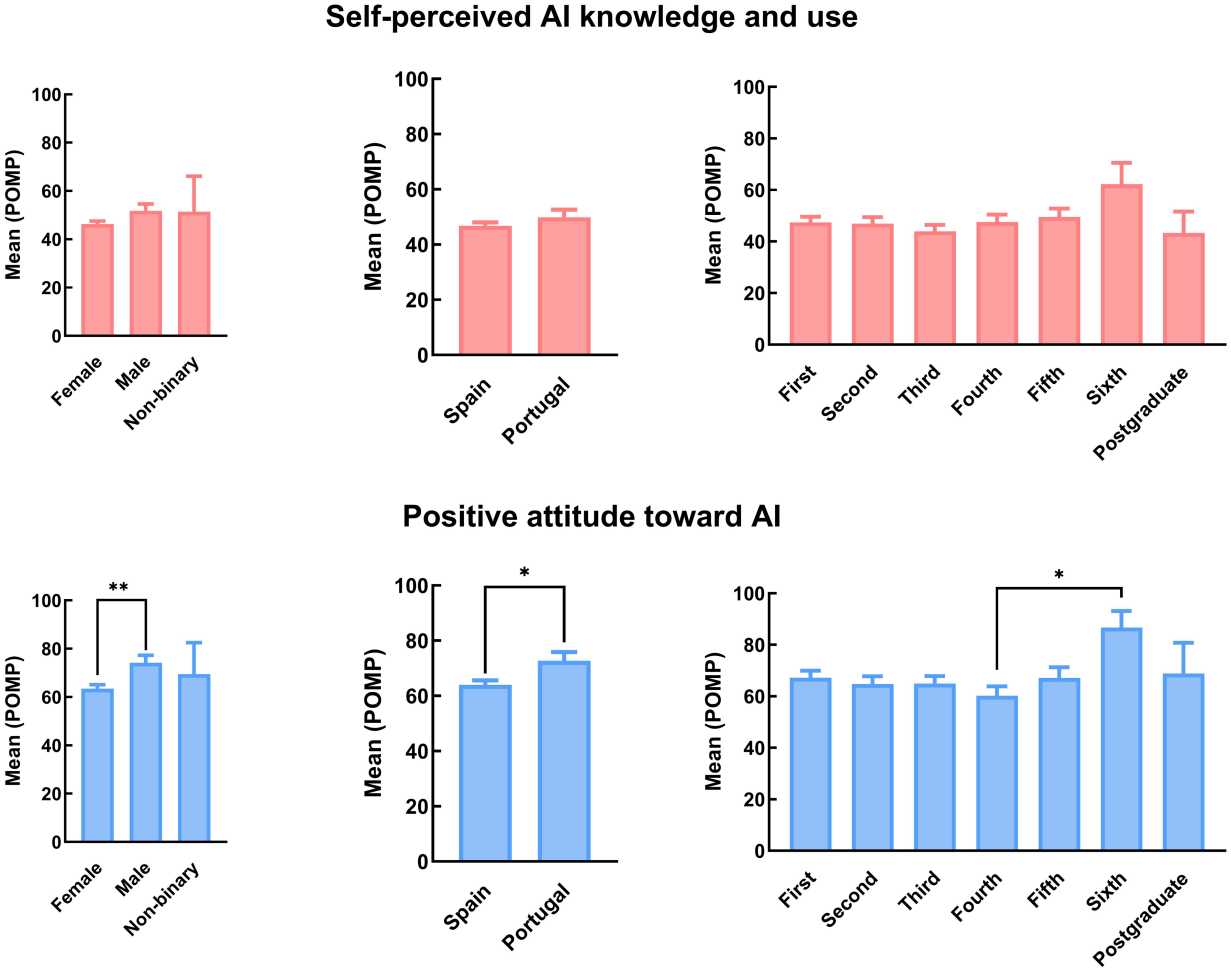
Self-perceived artificial intelligence (AI) knowledge and use (upper panels) and positive attitude toward AI (lower panels) according to sex (left), country (center), and academic year (right). Bars represent POMP-transformed means (percentage of maximum possible; 0–100 scale), and error bars indicate standard error of the mean (SEM). Group differences were analyzed using analysis of variance (ANOVA). Statistically significant differences between groups are indicated by connecting lines and asterisks (*p < 0.05; **p < 0.01).

To further explore potential sources of variability, comparisons were conducted according to sex, country, and academic level (Figure 3).

In the top panel, representing self-perceived AI knowledge and use (POMP scores), differences by sex, country, and academic level are displayed. With regard to sex, mean scores appear slightly higher among male and non-binary students compared to female students; however, no statistically significant differences were found for this comparison. When comparing countries, students from Portugal show marginally higher mean scores than those from Spain, although again no statistically significant difference was found. In contrast, differences across academic level reveal a clearer pattern: scores for self-perceived AI knowledge and use tend to increase in later years of the degree, with sixth-year students displaying the highest mean values, while postgraduate students and earlier-year students present comparatively lower scores. Although dispersion varies across groups, no statistically significant differences were found for academic level comparisons (Figure 3; pink bars).

In the bottom panel, representing positive attitudes toward AI (POMP scores), clearer statistically significant differences are observed. Regarding sex, male students show significantly higher attitude scores compared to female students (*p* < 0.01). Non-binary students present intermediate values, and no statistically significant pairwise differences were detected for this group. Interestingly, when comparing countries, Portuguese students demonstrate significantly higher positive attitude scores than Spanish students (*p* < 0.05). Across academic levels, statistically significant differences are also observed. Sixth-year students display the highest mean attitude scores, significantly exceeding those of at least one lower academic level group, as indicated. Earlier academic years, particularly the fourth year, show comparatively lower mean values (Figure 3; blue bars).

Overall, while self-perceived AI knowledge and use (top, pink) show descriptive variability without statistically significant differences across these sociodemographic factors, positive attitudes toward AI (bottom, blue) exhibit statistically significant differences by sex, country, and academic level, with higher scores observed among male students, Portuguese students, and those in the final year of the degree.

### 3.3 Distribution of prior AI training across institutions

Given the observed variability in self-perceived AI knowledge, use, and attitudes across institutions and sociodemographic groups, the next analysis examined students’ prior exposure to formal AI training.

**Figure 4:**
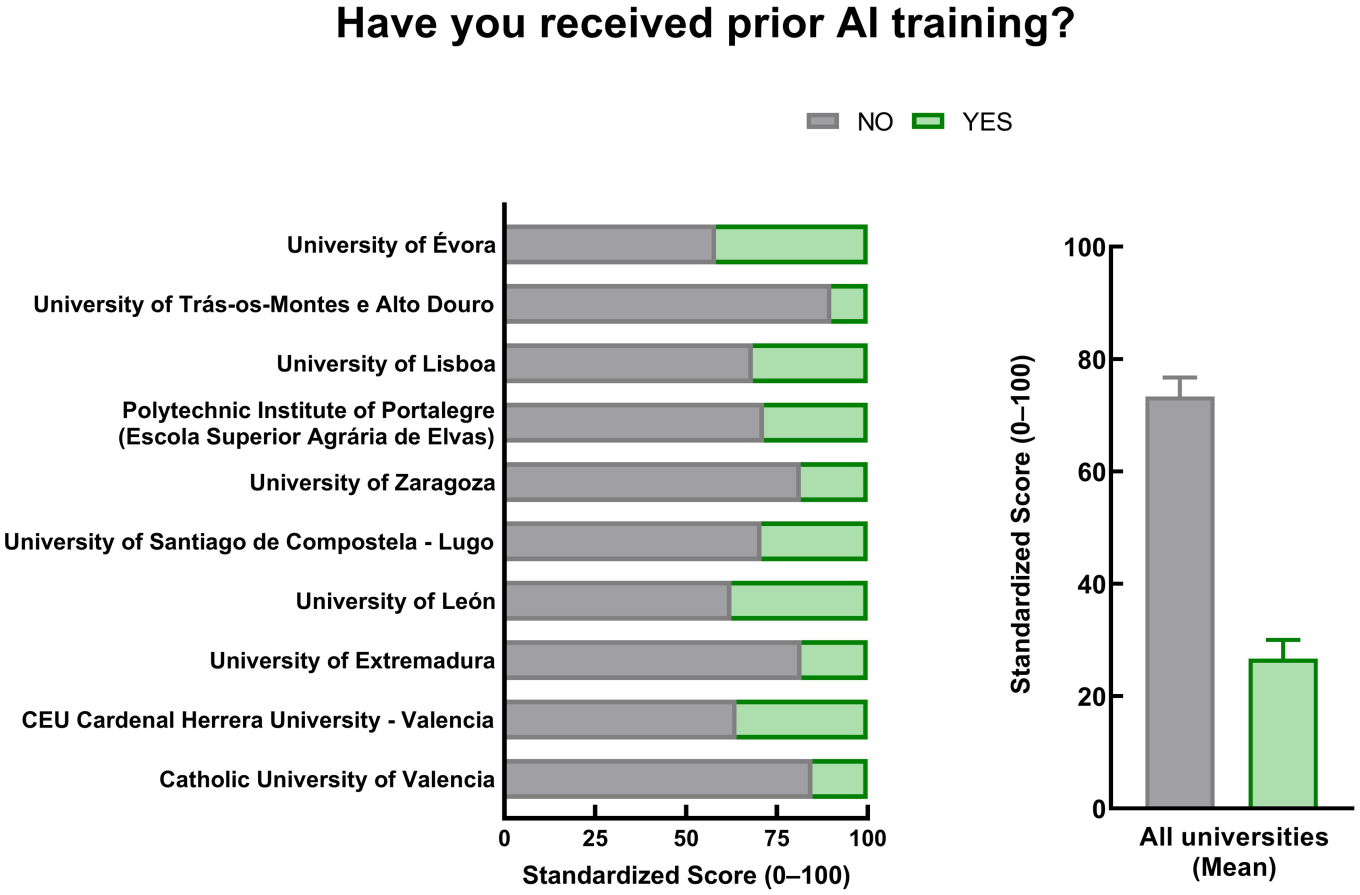
Self-reported prior training in artificial intelligence (AI) across participating institutions. Left panel shows the percentage of students reporting “Yes” or “No” to having received AI training at each university (standardized score, 0–100). Right panel presents the overall mean percentage across all institutions. Gray bars indicate students without prior AI training, and green bars indicate students reporting prior AI training. Error bars represent standard error of the mean (SEM).

Figure 4 presents the distribution of previous AI training (yes/no) across participating universities, expressed as standardized scores (0–100). The left panel displays the proportion of students reporting having received training (green) versus no training (grey) in each institution, while the right panel summarizes the overall mean across all universities.

Across institutions, the majority of students reported not having received prior AI training, as reflected in the figure. Although the proportion of trained students varied between universities, in all centers the percentage of students without prior AI training clearly exceeded that of those who reported having received training. At the aggregated level (right panel), the overall standardized mean confirms this pattern, with a substantially higher proportion of students indicating no prior training (73.4 ± 3.6) compared to those reporting previous AI education (26.7 ± 3.4).

Overall, the figure highlights that formal exposure to AI training remains limited across institutions, with notable inter-university variability but a consistent predominance of students lacking structured training in AI.

### 3.4 Type of AI training received

To further characterize the nature of AI exposure, a subsequent analysis focused specifically on those students who reported having received prior AI training.

**Figure 5:**
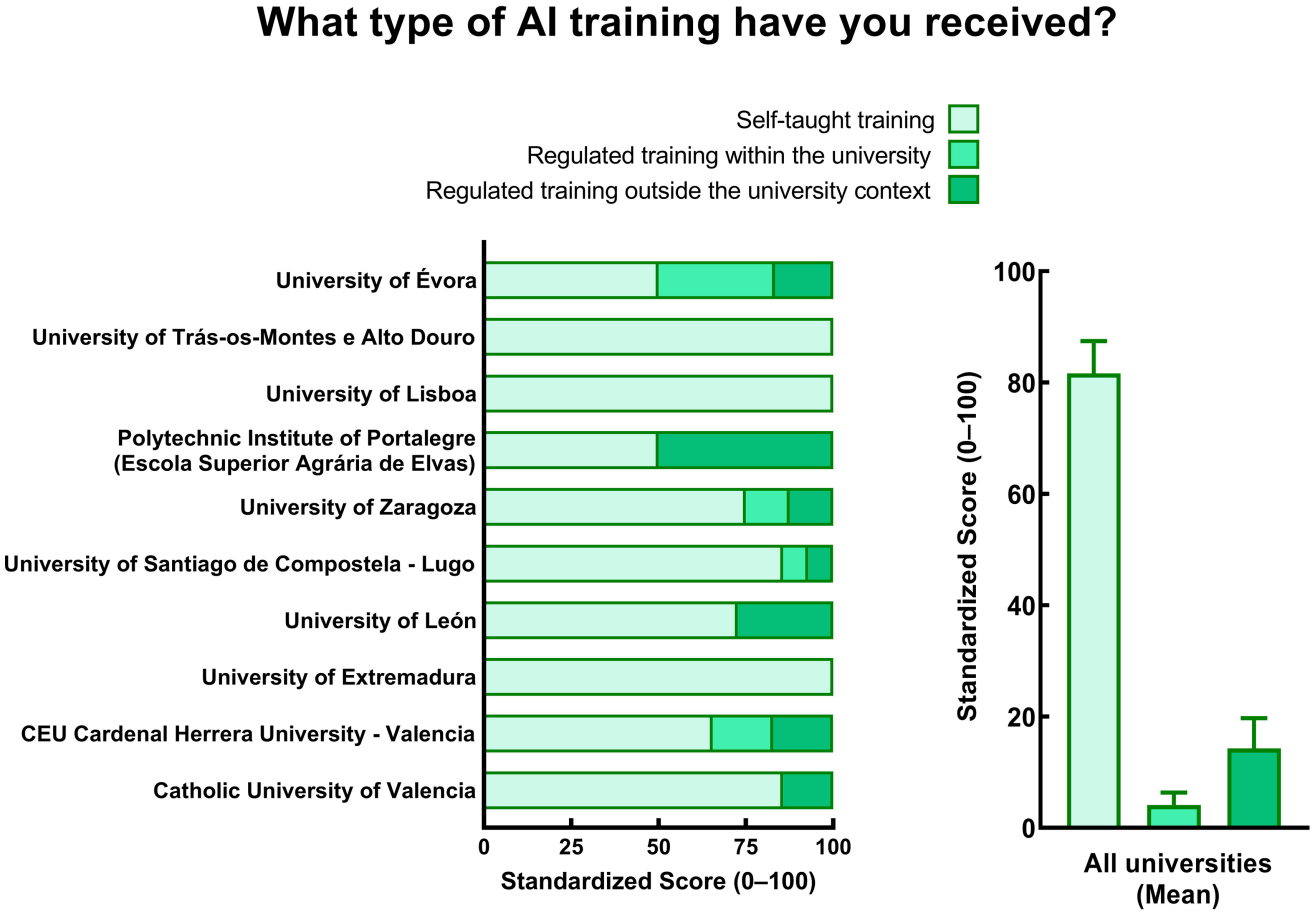
Type of artificial intelligence (AI) training reported by students who indicated prior exposure to AI. Left panel shows the distribution of training modalities across participating institutions (standardized score, 0–100). Training categories include self-taught training, regulated training within the university, and regulated training outside the university context. Right panel presents the overall mean percentage for each training modality across all institutions. Error bars represent standard error of the mean (SEM).

Figure 5 illustrates the type of training received among students who answered “yes” to having prior AI training. Training was classified into three categories: self-directed learning, regulated training within the university, and regulated training outside the university context. Results are presented as standardized scores (0–100), both by institution and as an overall mean across all universities.

Across institutions, self-directed training appears as the most prevalent form of AI learning among students reporting prior exposure. In contrast, regulated training outside the university context, primarily corresponding to structured education received during secondary schooling, shows intermediate but highly variable representation across centers, whereas regulated training within the university context is comparatively less frequent. Notably, in three universities, students reported no regulated AI training within the university setting at all, indicating a complete absence of institutionally embedded AI instruction in those centers, where any AI-related learning appears to depend primarily on students’ own initiative.

At the aggregated level (right panel), the overall mean confirms this pattern, with self-directed training representing the largest proportion by far, followed by regulated training outside the university, and finally regulated training within the university as the least frequent modality.

Overall, these findings suggest that when AI training occurs, it is predominantly acquired through informal or self-initiated learning rather than through structured institutional programs.

### 3.5 Association between prior AI training and self-perceived AI knowledge, use, and attitudes

Having characterized the prevalence and type of AI training, the next analysis examined whether prior exposure to any form of AI training was associated with differences in students’ self-perceived AI-related knowledge, use, and attitudes.

**Figure 6:**
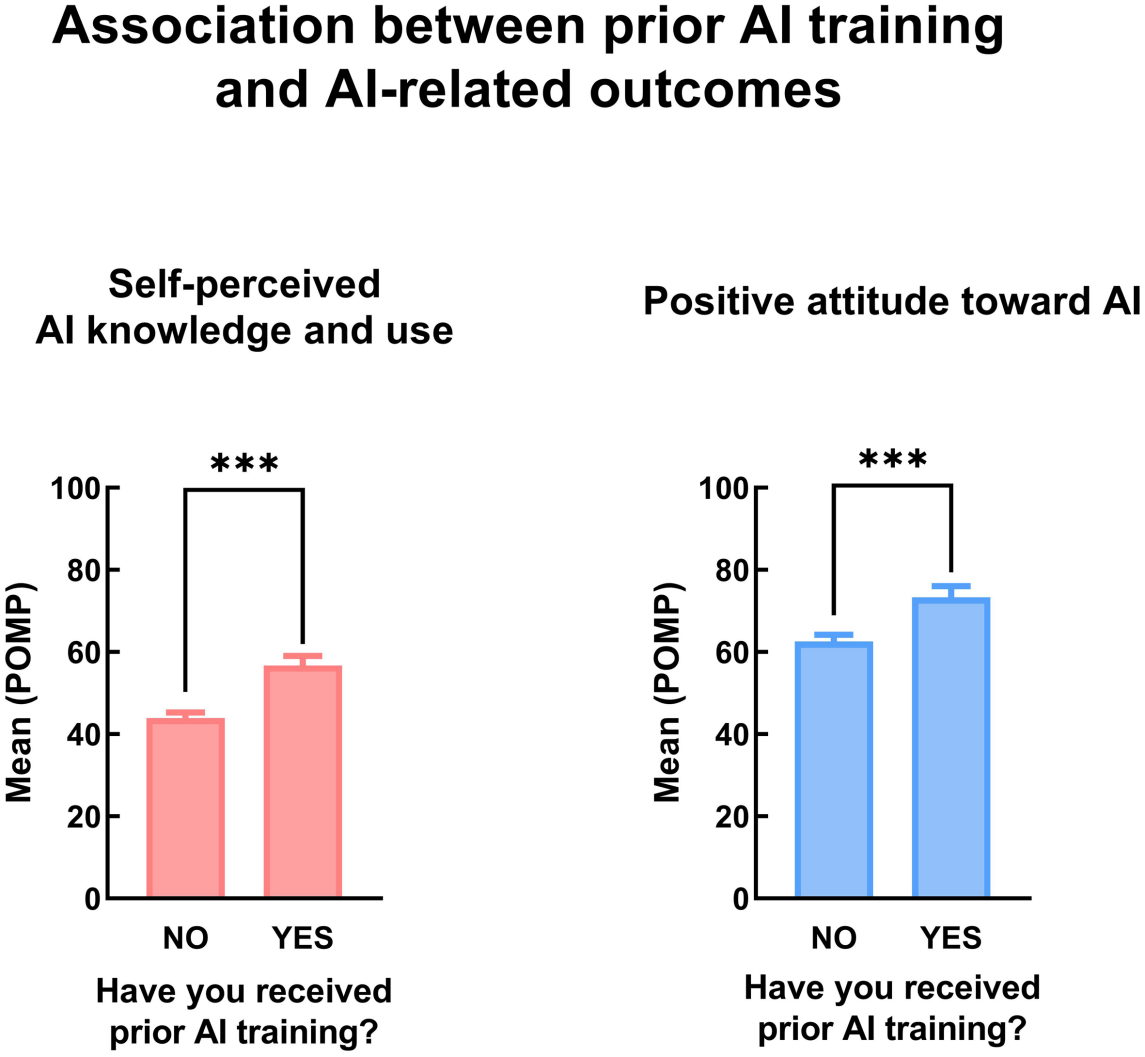
Association between prior artificial intelligence (AI) training and AI-related outcomes. Left panel shows POMP-transformed mean scores (percentage of maximum possible; 0–100 scale) for self-perceived AI knowledge and use, and right panel shows POMP-transformed mean scores for positive attitude toward AI, according to whether students reported prior AI training (“Yes” or “No”). Error bars represent standard error of the mean (SEM). Differences between groups were analyzed using independent samples *t*-tests. Statistically significant differences are indicated by connecting lines and asterisks (***p < 0*.001*).

Figure 6 compares students who reported having received any type of AI training with those who had not. Results indicate that students with prior AI training show higher scores in self-perceived AI knowledge and use, particularly in relation to the reported use of AI-based applications in academic contexts and awareness of AI’s potential in both educational and professional veterinary settings. These differences are statistically significant (*p* < 0.001), indicating a clear association between prior training and greater engagement with AI tools.

Similarly, prior AI training is associated with more positive attitudes toward AI. Students who had received training reported significantly higher agreement with statements regarding AI’s potential to improve veterinary education and its anticipated impact on the veterinary profession. They also reported feeling more prepared to work with AI tools in their future professional activity. In contrast, students without prior AI training consistently displayed lower scores across these dimensions (*p* < 0.001).

To further quantify the magnitude of these differences, an effect size analysis was conducted (Table 2). For self-perceived AI knowledge and use, a moderate-to-large effect size was observed (Cohen’s d = −0.62; 95% CI [−0.87, −0.37]), indicating that students without AI training scored substantially lower than those who had received training. For positive attitude toward AI, the effect size was moderate (Cohen’s d = −0.41; 95% CI [−0.66, −0.17]). According to Cohen’s benchmarks (19), these values reflect practically meaningful differences, reinforcing the association between prior AI training and higher AI-related outcomes.

**Table 2:** Effect size analysis for differences in AI-related outcomes according to prior AI training.

AI-related outcome	Effect size measure	Standardizer	Point estimate	95% CI lower	95% CI upper
Self-perceived AI knowledge and use	Cohen’s d	3.6947	−0.6213	−0.8713	−0.3704
Positive attitude toward AI	Cohen’s d	2.3436	−0.4145	−0.6620	−0.1665

Independent-samples comparisons were conducted between students who reported prior AI training and those who did not. Cohen’s d values and 95% confidence intervals (CI) are presented for self-perceived AI knowledge and use and positive attitude toward AI. Negative values indicate lower scores among students without prior AI training. Effect sizes are interpreted according to conventional benchmarks.

Overall, the results suggest that prior exposure to AI training, regardless of its modality, is associated with higher levels of AI use, greater awareness of its academic and professional applications, and more favorable attitudes toward its integration.

### 3.6 Association between daily social media use and self-perceived AI knowledge, use, and attitudes

**Figure 7:**
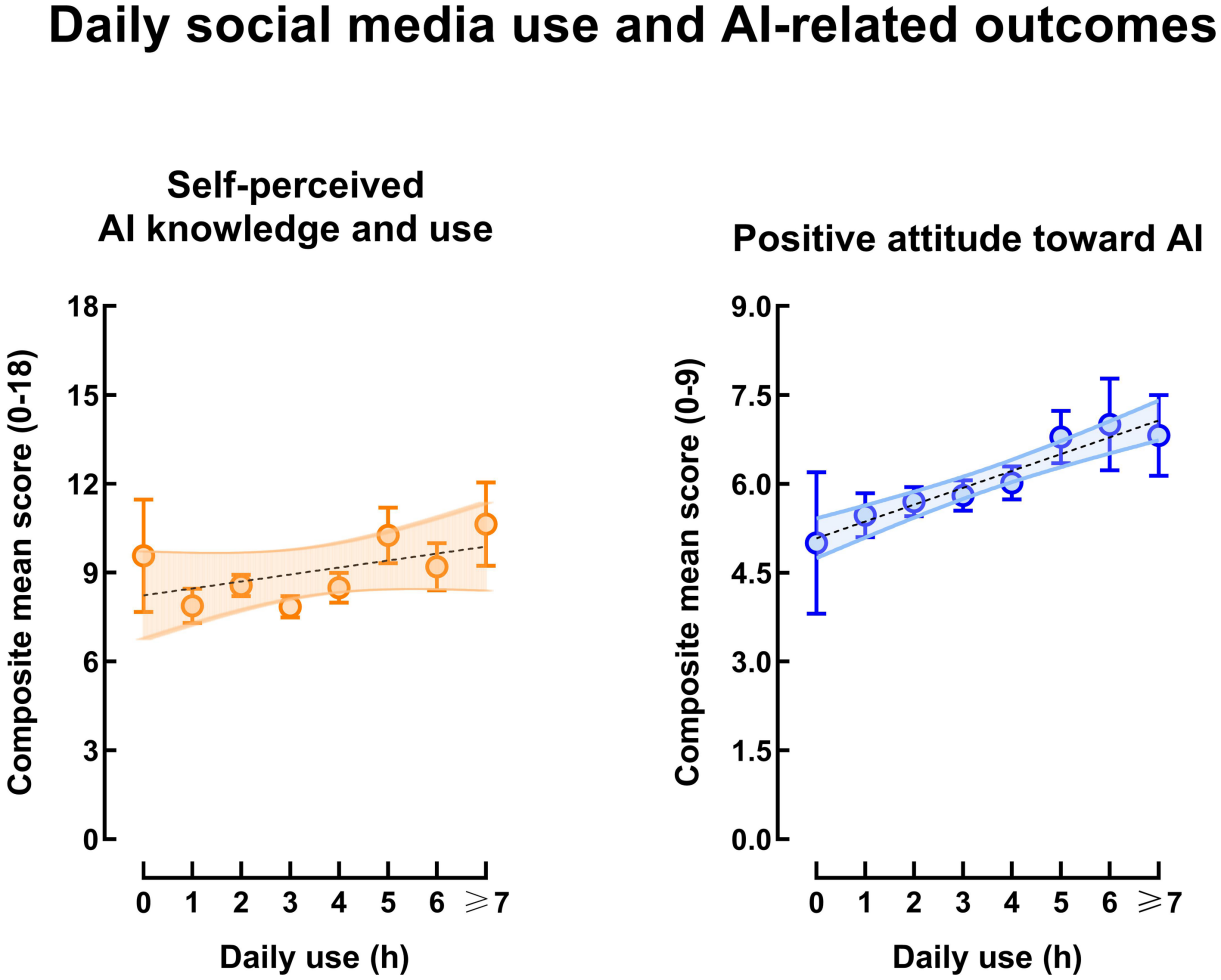
Association between daily social media use and artificial intelligence (AI)-related outcomes. Left panel shows composite mean scores for self-perceived AI knowledge and use (0–18 scale), and right panel shows composite mean scores for positive attitude toward AI (0–9 scale), according to self-reported daily social media use (hours per day). Points represent group means, and error bars indicate standard error of the mean (SEM). Dashed lines represent linear trend lines. Pearson correlation analyses were performed to assess the association between daily social media use and AI-related outcomes.

Given that informal digital exposure may influence familiarity with emerging technologies, the association between daily social media use and AI-related outcomes was examined (Figure 7; Table 3).

**Table 3:** Pearson correlation between daily social media use and AI-related outcomes.

AI-related outcome	Statistic	Value
Self-perceived AI knowledge and use	Pearson correlation	0.115*
	Two-tailed *p*-value	0.0343
	*N*	336
Positive attitude toward AI	Pearson correlation	0.157**
	Two-tailed *p*-value	0.0037
	*N*	338

Pearson correlation coefficients (r), two-tailed p-values, and sample sizes (N) are shown for the association between daily social media use (hours per day) and self-perceived AI knowledge and use, as well as positive attitude toward AI. Asterisks indicate statistical significance: *p < 0.05; **p < 0.01.

As illustrated in Figure 7, a progressive increase in self-perceived AI knowledge and use scores is observed as daily social media use increases. Students reporting no or minimal daily use show comparatively lower composite mean scores (raw scale 0–18), whereas those in the higher-use categories tend to display higher values. Although some fluctuation is present across intermediate categories, the overall pattern suggests a positive relationship between intensity of social media use and self-perceived AI knowledge and use.

This descriptive trend is supported by the correlation analysis presented in Table 3, which shows a statistically significant positive Pearson correlation between daily social media use and self-perceived AI knowledge and use (r = 0.115, *p* = 0.0343). Although the magnitude of the correlation is small, it indicates that higher levels of daily social media exposure are associated with greater reported knowledge and use of AI tools.

A similar descriptive pattern was observed for positive attitudes toward AI, as shown in the right panel of Figure 7. Attitude scores progressively increased (raw composite scale 0–9) across categories of daily social media use, with the highest values observed among students reporting six or more hours per day. The correlation analysis was consistent with this pattern, revealing a statistically significant positive relationship between daily social media use and positive attitudes toward AI (r = 0.157, *p* = 0.0037). As with self-perceived AI knowledge and use, the effect size was modest but statistically significant.

Overall, both the graphical trends and the correlation analysis indicate that higher daily engagement with social media is positively associated with self-perceived AI knowledge and use and, more consistently, with more favorable attitudes toward AI.

### 3.7 Categorized social media use and its association with self-perceived AI knowledge and attitudes

**Figure 8:**
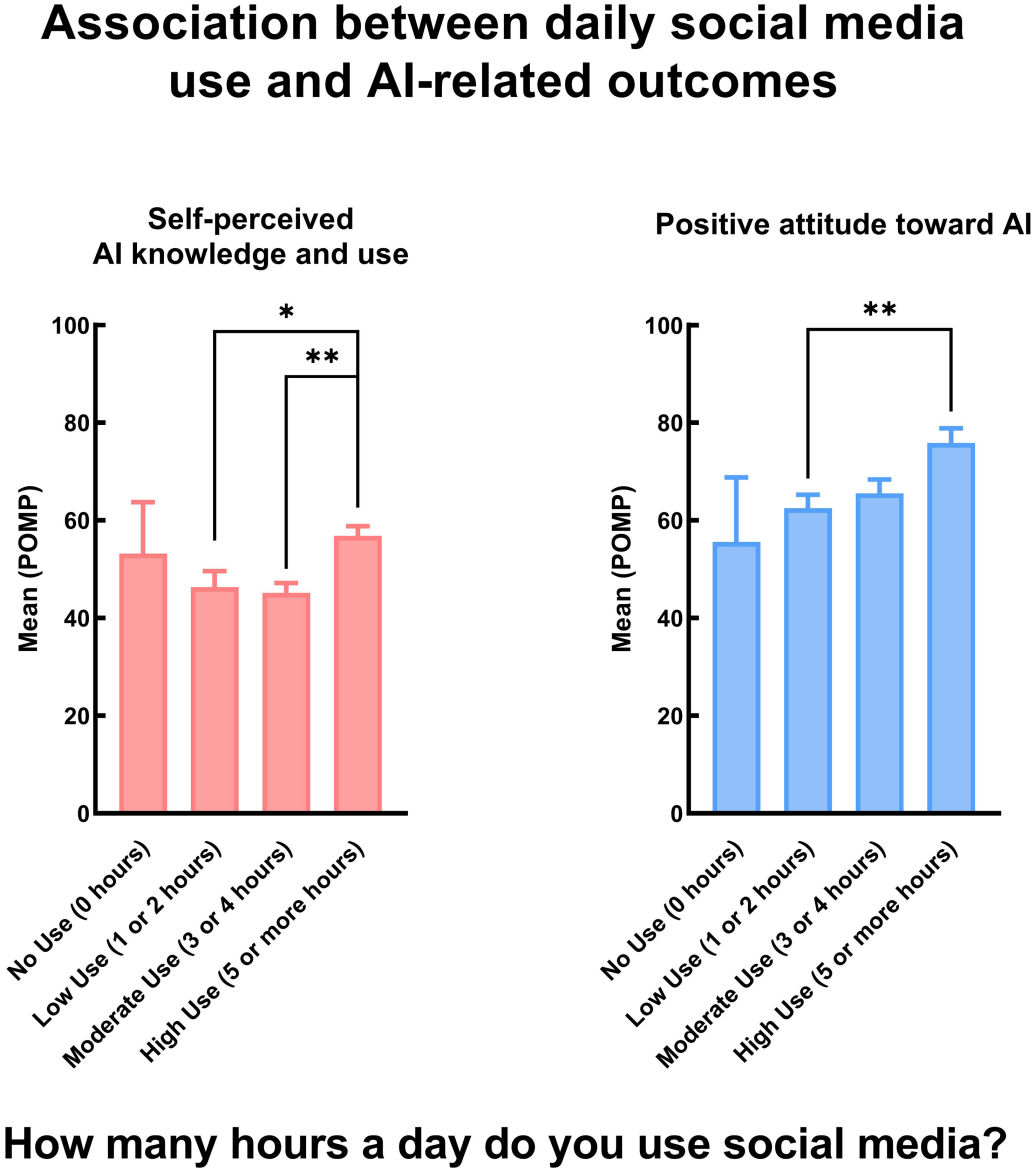
Association between daily social media use and artificial intelligence (AI)-related outcomes. Left panel shows POMP-transformed mean total scores (percentage of maximum possible; 0–100 scale) for self-perceived AI knowledge and use, and right panel shows POMP-transformed mean total scores for positive attitude toward AI, according to daily social media use categories (no use: 0 h; low use: 1–2 h; moderate use: 3–4 h; high use: ≥5 h). Bars represent mean ± standard error of the mean (SEM). Group differences were analyzed using one-way analysis of variance (ANOVA), followed by post hoc pairwise comparisons. Differences between groups are indicated by connecting lines and asterisks (*p < 0.05; **p < *0.01*).

To further explore the relationship between digital exposure and AI-related outcomes, daily social media use was categorized into four groups (no use: 0 h; low use: 1–2 h; moderate use: 3–4 h; high use: ≥5 h), and differences in self-perceived AI knowledge, use, and attitudes were examined (Figure 8).

In the top panel (pink bars), representing self-perceived AI knowledge and use (POMP scores), differences between categories are observed. Students classified as high social media users show the highest mean scores, whereas moderate and low users display comparatively lower values. Non-users present intermediate scores, though below those of high-use students. Statistical markers indicate significant differences between specific categories, particularly between high users and lower-use groups, suggesting that more intensive social media engagement is associated with greater self-perceived AI knowledge and reported use.

In the bottom panel (blue bars), representing positive attitudes toward AI, a clearer gradient is observed. Mean attitude scores increase progressively from non-users to high users. High social media users display the highest attitude scores, and statistically significant differences are indicated between high users and at least one lower-use category. This pattern reinforces the positive association previously observed in the correlation analysis.

Overall, the categorized analysis was consistent with the findings from Figure 7 and Table 3, indicating that higher levels of social media engagement are associated with higher self-perceived AI knowledge and use, together with more positive attitudes. The convergence between the categorical ANOVA results and the Pearson correlation analysis provides descriptive support for this weak but statistically significant association.

## 4 Discussion

The present study provides a cross-institutional overview of veterinary students’ self-perceived knowledge, use, and attitudes toward artificial intelligence (AI) in Spain and Portugal. Three main findings emerged. First, there is clear variability between institutions in self-perceived AI knowledge and attitudes. Second, prior AI training is associated with higher self-perceived knowledge, greater use, and more positive attitudes. Third, daily digital engagement, especially social media use, is positively related to AI-related outcomes. Overall, these findings suggest that exposure to AI, whether formal or informal, plays an important role in shaping students’ readiness to engage with these technologies.

One of the most relevant results is the marked variability observed between institutions. Students’ exposure to and familiarity with AI are not consistent across veterinary schools, even in countries with similar higher education systems. This finding is consistent with previous research showing that AI integration in higher education is uneven and depends on institutional strategies, faculty involvement, and policy decisions (20, 21). In this sense, the institutional context appears to influence how students experience and understand AI.

Some institutions reported no regulated AI training within the university context. This supports the idea that curricular integration of AI is still inconsistent, as described in other higher education and health-related disciplines (1, 5). In these settings, students may rely more on self-directed learning or external sources rather than structured academic instruction, a pattern also described in previous literature (6). In contrast, institutions with higher levels of self-perceived AI knowledge and use may reflect greater curricular exposure or stronger institutional engagement with digital innovation (5, 16).

However, these differences must be interpreted with caution. The cross-sectional design of this study does not allow causal conclusions. Differences between institutions cannot be attributed only to formal training, as AI adoption in education is influenced by multiple factors, including institutional culture, available resources, and strategic priorities (1). Research in health professions education also suggests that attitudes and preparedness toward AI are shaped not only by formal teaching but also by broader social and organizational environments (16). Even so, the overall pattern indicates that institutional context likely plays a meaningful role in students’ AI preparedness.

These findings are particularly relevant given the growing presence of AI in healthcare. AI systems are increasingly used in clinical decision-making and diagnostics (6, 22). Despite this, many veterinary programs still lack structured AI or machine learning training, even though students express strong interest in these topics (23). Similar concerns have been raised in medical education, where scholars call for formal AI integration to ensure that graduates can use these tools responsibly and critically (6, 24, 25). Differences in institutional exposure may therefore influence students’ confidence and perceived readiness.

A second key finding concerns the association between prior AI training and AI-related outcomes. Students who reported any type of AI training —whether self-directed, university-based, or external—showed higher self-perceived knowledge, greater use, and more positive attitudes. This is consistent with findings in veterinary education showing that students with previous AI experience report higher engagement and perceive AI-supported activities as useful (26). Similarly, studies in medical education indicate that practical exposure to AI is associated with more positive perceptions of its professional relevance (27). Veterinary students have also expressed strong interest in formal AI integration (9, 23). Evidence from course-based AI activities further suggests that guided and structured use may promote more critical engagement than purely individual experimentation (26, 28).

At the same time, these associations should not be interpreted as causal, primarily because it is impossible to determine whether training enhances self-perceived AI knowledge and attitudes, or whether students who are already more interested in technology are more likely to pursue such training. In fact, both scenarios are plausible and not mutually exclusive. Nevertheless, the consistency of these associations across multiple dimensions suggests that structured exposure is meaningfully related to AI readiness.

Beyond formal training, informal digital engagement also appears relevant. In this study, higher daily social media use was positively associated with self-perceived AI knowledge, use, and especially attitudes. Although these correlations were modest, they were statistically significant and directionally consistent. Previous research shows that digital engagement can influence how students perceive emerging technologies (20, 21). In our data, the association was stronger for attitudes than for self-perceived knowledge, suggesting that digital exposure may influence acceptance and perception more than structured competence. Again, causality cannot be inferred.

From an educational perspective, these findings have clear implications. Structured AI exposure is associated with more positive outcomes, yet formal training remains limited in many programs (23). Scholars in educational health professions argue that AI should be integrated into curricula to support responsible and critical use (6). Institutions are also encouraged to adapt assessment and governance frameworks to the growing presence of AI (20). This becomes even more relevant considering that large language models can achieve competitive results in medical licensing examinations (29) and high accuracy in veterinary multiple-choice assessments (30).

However, it is important to avoid assuming that AI automatically improves learning. Some controlled studies show that students using AI tools do not necessarily perform better than those working independently, even if they perceive the tools as useful (4, 9). This highlights the need for pedagogically guided integration rather than unstructured reliance.

Interestingly, the patterns observed in the Iberian context do not appear substantially different from those reported in Australia, the United States, and other international settings, where high levels of informal AI use coexist with limited structured training and strong student interest in curricular integration (9, 23, 31). This suggests that the challenges identified here may reflect broader international trends rather than region-specific gaps.

In summary, this study offers a cross-institutional perspective on how veterinary students are currently engaging with AI in the Iberian Peninsula. It shows variability across institutions, associations between training and AI-related outcomes, and links between digital engagement and attitudes. While previous research has often focused on technical performance or isolated interventions, this study provides broader evidence on students’ exposure and perceptions.

Several limitations should be considered when interpreting these findings. First, AI knowledge was assessed through self-reported questionnaire responses and should therefore be interpreted as self-perceived knowledge rather than objective technical competence. Second, sex-related differences in positive attitudes toward AI should be interpreted cautiously because the sample was predominantly female, reflecting the current demographic composition of veterinary education but resulting in unequal group sizes. Third, responses to items addressing ethical use of AI, cheating, plagiarism, and prohibition of AI tools may have been influenced by social desirability bias, as students may have preferred answers perceived as academically or ethically appropriate. Finally, the absence of a neutral midpoint may have encouraged participants with no clearly formed opinion to select a directional response, which should be considered when interpreting the distribution of attitudes.

Despite these limitations, the findings highlight that veterinary education is at a turning point. Students are already using AI in their academic work, often informally, but institutional integration remains uneven. The central question is no longer whether AI will influence veterinary education, but how institutions will respond. Preparing future veterinarians to use AI critically, ethically, and responsibly should be considered a priority for the coming years.

## Data Availability

The raw data supporting the conclusions of this article will be made available by the authors, without undue reservation.
